# Osteofibrous dysplasia-like adamantinoma: A case report and literature review

**DOI:** 10.3389/fonc.2022.967294

**Published:** 2022-11-11

**Authors:** Jian-Wei Li, Lei Miao, Zhen-Guo Zhao, Lin Yang, Zhuo Shi, Meng Li

**Affiliations:** ^1^ Department of Diagnostic Radiology, National Cancer Center/ National Clinical Research Center for Cancer/Cancer Hospital, Chinese Academy of Medical Sciences and Peking Union Medical College, Beijing, China; ^2^ Department of Orthopaedics, National Cancer Center/National Clinical Research Center for Cancer/Cancer Hospital, Chinese Academy of Medical Sciences and Peking Union Medical College, Beijing, China; ^3^ Department of Pathology, National Cancer Center/National Clinical Research Center for Cancer/Cancer Hospital, Chinese Academy of Medical Sciences and Peking Union Medical College, Beijing, China

**Keywords:** bone tumor, osteofibrous dysplasia-like adamantinoma, plain radiography, computed tomography, magnetic resonance imaging

## Abstract

**Abstract background:**

Osteofibrous dysplasia-like adamantinoma (OFD-like adamantinoma), classical adamantinoma and dedifferentiated adamantinoma were previously considered to be three subtypes of adamantinoma of long bones. In the 5th edition of the World Health Organization (WHO) classification of bone tumors in 2020, OFD-like adamantinoma was newly proposed and classified as an intermediate-locally aggressive tumor in other mesenchymal tumors of bone. OFD-like adamantinoma is rare, accounting for only 0.4% of all primary bone tumors. OFD-like adamantinoma is often misdiagnosed due to the insufficient understanding of it. Here we report a case of OFD-like adamantinoma treated in our hospital with a literature review.

**Case presentation:**

The patient, a 14-year-old male, had swelling in his right leg with intermittent pain for one year. Plain radiography, computed tomography (CT) and magnetic resonance imaging (MRI) were performed. Based on the radiological and histological examinations, a diagnosis of OFD-like adamantinoma was suspected. After admission, the patient underwent tumor resection of the right tibia, free transplantation of the left fibula and internal fixation. After resection of the tumor, the wound recovered well, the vital signs were stable, and activity was normal. The patient has been followed up for more than a year with no recurrence or distant metastasis.

**Conclusion:**

OFD-like adamantinoma is a rare primary bone tumor with nonspecific clinical features. Imaging examination can demonstrate the lesion and help diagnosis. The pathological discovery of epithelioid tissue is the key evidence for diagnosis.

## Introduction

Osteofibrous dysplasia-like adamantinoma, which is relatively rare to encounter in clinical practice, is also known as OFD-like adamantinoma and is a kind of low-grade malignant bone tumor that is different from classical adamantinoma and osteofibrous dysplasia (OFD). A case of OFD-like adamantinoma treated in our hospital is reported in this paper with a literature review.

## Case description

**Figure 1:**
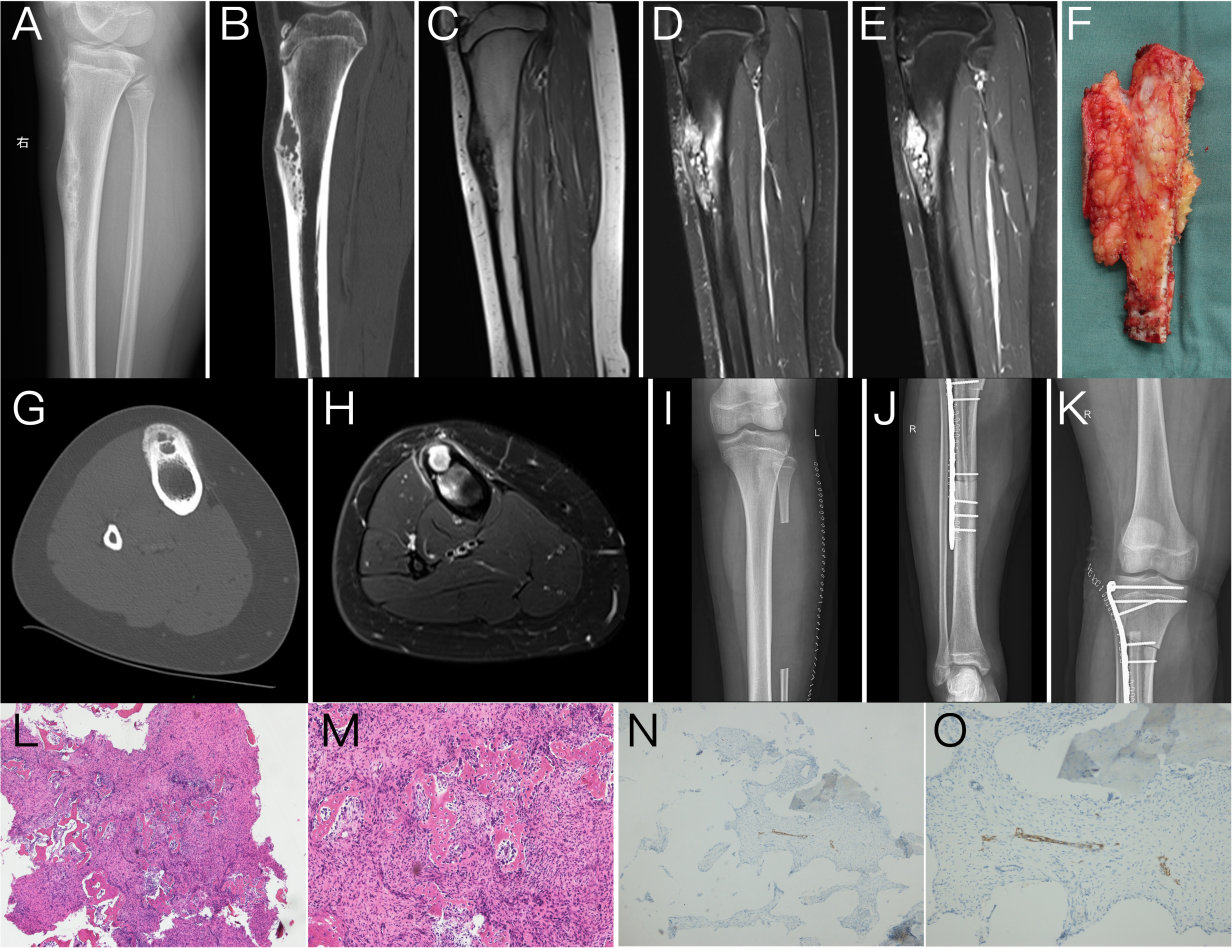
Lateral plain radiography of the tibia **(A)** showed a polycystic expansive bone destruction area in the anterior cortex of the upper segment of the right tibia with a sclerotic edge that was irregular and lobulated, and the long axis of the lesion was consistent with the tibia. The sagittal reformation image of CT **(B)** and the axial image of CT **(G)** clearly showed that the lesion is an eccentric polycystic expansive transparent area while the bone cortex expands and thins. Cortical interruption could be seen in some layers with a small periosteal reaction in some parts, and cortical hyperplasia, sclerosis, and cortical destruction could be visualized around the lesions. MRI showed abnormal signal lesions of the upper tibia on the right side with low signal intensities on T1-weighted images (T_1_WI) **(C)** and high signal intensities on sagittal and axial T2-weighted images (T_2_WI) **(D, H)**. The lesions were significantly enhanced on postcontrast scans **(E)**. The postoperative plain radiography of the lower limb **(I–K)** showed postoperative changes of tumor resection of the right tibia, free transplantation of the left fibula, and internal fixation. The gross specimen during the operation **(F)** showed a sagittal view of the tumor and the tibia, which had a good comparison with the lateral plain radiography and sagittal CT and MRI images **(A–E)**. Tumor pathological HE sections **(L, M)** showed irregularly woven bone trabeculae surrounded by osteoblasts, with many fibrous matrix and small nests of epithelial cells. Immunohistochemistry **(N, O)** showed nests of small tumor cells positive for cytokeratin CK19.

A 14-year-old male patient had swelling in his right leg with intermittent pain for 1 year. The swelling of the lower leg was severe after exercise and could be relieved by rest. The previous month, he was treated at an external hospital, and the plain film radiography showed a lesion of the right tibia, so he came to our hospital for treatment. Physical examination: both lower limbs were equal in length, and obvious swelling or tenderness was not palpated in the right lower leg. The movement of both lower limbs and the muscle strength were normal. Plain radiography: the cortical expansive bone destruction of the anterior edge of the right upper tibia was irregular and lobulated, and the sclerotic edge could be seen. The long axis of the lesion was consistent with the tibia, with a maximum cross-sectional area of 2.1 × 1.9 cm and a length of approximately 7.5 cm, with no obvious periosteal reaction and no definite soft tissue mass. Computed tomography (CT): the lesion showed an eccentric polycystic expansive lucency area, and the bone cortex was expanded and thinned. Cortical interruption could be seen on some levels, and a small local lamellar periosteal reaction was observed in some parts. The lesion was associated with pathological fracture. Magnetic resonance imaging (MRI): the lesion of the upper right tibia showed a low signal on T1-weighted imaging (T_1_WI), a high signal on T2-weighted imaging (T_2_WI), and a high signal on diffusion-weighted imaging (DWI). The lesion showed significant enhancement on the postcontrast MRI scan. The preoperative imaging diagnosis was OFD of the upper right tibia with pathological fracture. After admission, the patient underwent tumor resection of the right tibia, free transplantation of the left fibula, and internal fixation. During the operation, the lesion was found to be located in the anterolateral bone cortex of the right tibia, and the tumor was white, flexible, and bleeding. The patient had good tolerance to the surgery with stable intraoperative condition. After resection of the tumor, the wound recovered well, the vital signs were stable, and activity was normal. Pathological examination showed a small amount of hyperplastic fibrous tissue and irregular woven bone and osteoblasts, in which scattered nest-like epithelial cells were seen. The immunohistochemistry results showed Ki-67 (+1%), SATB2 (+), CK19 (1+), AE1/AE3 (1+), CK18 (−), EMA (−), P63 (1+), S-100 (−), MDM2 (2+), SMA (−), and β-catenin (2+, cytoplasm) (**Figure 1**). The patient was recommended to be closely followed up and observed after the operation, avoiding weight bearing to prevent the aggravation of pathological fracture. The patient has been followed up for more than a year with no recurrence or distant metastasis.

## Discussion

In addition to odontogenic epithelial tumors of the jaw, adamantinoma of long bones is the only truly primary epithelial tumor of bone. OFD-like adamantinoma, classical adamantinoma, and dedifferentiated adamantinoma were previously considered to be three subtypes of adamantinoma of long bones. In the 5th edition of the World Health Organization (WHO) classification of bone tumors in 2020, OFD-like adamantinoma was newly proposed and classified as an intermediate-locally aggressive tumor in other mesenchymal tumors of bone, while adamantinoma of long bones and dedifferentiated adamantinoma were classified as malignant tumors in other mesenchymal tumors of bone (1, 2). OFD-like adamantinoma is rare, accounting for only 0.4% of all primary bone tumors (3).

OFD-like adamantinoma grows slowly, with an average onset at 14 years of age, and there is no significant sex difference (4). Most patients experience clinical manifestations of swelling, pain, or pathological fracture (5). Under the pathological microscope, osteoblasts surround the bone fibrous stroma with needle-woven bone, which is characterized by nested epithelial cell clusters or scattered isolated epithelial cells under the background of OFD. Immunostaining can show cytokeratin (CK) positivity. The diagnosis should be further confirmed by immunohistochemistry (6–8). In this case, CK14 and AE1/AE3 were expressed in the tumor, suggesting that it was derived from epithelial tissue.

The location of OFD-like adamantinoma is characteristic and is mostly located in the shaft of the tibia (7). It is an intracortical lesion that occurs especially in the anterior cortex. Keeney et al. found that some sample cases (approximately 13%) were associated with ipsilateral fibular lesions (9). OFD-like adamantinoma is a locally invasive bone tumor with both benign and malignant features. Plain radiography shows eccentric cystic expansive bone destruction, which can be osteolytic or osteolytic sclerotic mixed type. The edge can be more often irregular. Periosteal reactions and soft tissue masses are rare, and anterior tibial anterior tibial bowing can be seen. Compared with plain radiography, CT, especially on thin layers with multiplanar reformation (MPR) reconstruction, can better show the details of cortical destruction and soft tissue density in the area of bone destruction and help detect subtle pathological fractures. The MRI signal is nonspecific. Soft tissue in the damaged area shows a slightly low signal on T_1_WI and an uneven high signal on T_2_WI, and the postcontrast scan shows heterogeneous enhancement. The superiority of MRI can better reflect the cystic and solid components of the tumor and the involvement of the medullary cavity, soft tissue, and the surrounding edema. The imaging examination of this case was comprehensive: the plain radiography, CT, and plain/postcontrast MRI findings were basically consistent with the imaging features of OFD-like adamantinoma. In this case, a small periosteal reaction was observed, which may be related to pathological fracture.

The histological and imaging features of OFD, OFD-like adamantinoma, and adamantinoma of long bones are closely related. They were previously considered to belong to the same disease pedigree (10), but it is difficult to distinguish them. OFD, also known as ossifying fibroma, is a benign disease whose age of onset, location, and imaging features are similar to those of OFD-like adamantinoma. It is often difficult to distinguish these conditions by imaging methods alone (11). Based on preoperative imaging, this case was diagnosed as the more common OFD, and the diagnosis of OFD-like adamantinoma was confirmed only after pathological microscopic observation and immunohistochemical detection of epithelial components. Adamantinoma of long bones is a low-grade malignant tumor. Compared with the features of OFD, invasive/malignant features such as the longitudinal length of the lesion, involvement of the medullary cavity, and moth-eaten margins may indicate a diagnosis of OFD-like adamantinoma (10). Some studies have shown that OFD-like adamantinoma can progress to adamantinoma. Hatori et al. speculated that OFD-like adamantinoma may be the precursor lesion of adamantinoma of long bones, but no research has confirmed this (3). Two cases of newly diagnosed OFD-like adamantinoma reported by Hazelbag in 1994 developed into adamantinoma of long bones when they recurred locally (12). Retrospective analysis found that there was a possibility of misdiagnosis by needle biopsy histology at the first diagnosis, which suggested that a biopsy of the lesion should be taken extensively and from multiple sites, and immunohistochemistry should be added to aid in the diagnosis when necessary. OFD-like adamantinoma has a better outcome than classic adamantinoma, but long-term follow-up is necessary due to the possibility of local recurrence and late complications (13).

In conclusion, OFD-like adamantinoma is rare, and its clinical features are not specific. Imaging examination can demonstrate the lesion well and provide valuable help for diagnosis and differential diagnosis, but the final diagnosis often requires multidisciplinary consultation (14). The pathological discovery of epithelioid tissue is the key evidence for diagnosis.

## Data availability statement

The original contributions presented in the study are included in the article/supplementary material. Further inquiries can be directed to the corresponding author.

## Ethics statement

The study involving human participants was reviewed and approved by Cancer Hospital, Chinese Academy of Medical Sciences. Written informed consent requirements were waived due to the retrospective character of this study.

## Author contributions

Z-GZ, LM, ZS, ML performed the data acquisition. ML, J-WL, LM performed the radiological images analysis. LY performed the histological analysis. ML, J-WL, LM performed the manuscript preparation. All authors contributed to the article and approved the submitted version.

## Conflict of interest

The authors declare that the research was conducted in the absence of any commercial or financial relationships that could be construed as a potential conflict of interest.

## Publisher’s note

All claims expressed in this article are solely those of the authors and do not necessarily represent those of their affiliated organizations, or those of the publisher, the editors and the reviewers. Any product that may be evaluated in this article, or claim that may be made by its manufacturer, is not guaranteed or endorsed by the publisher.
